# Ultrasensitive Label-Free Detection of Free Thyroxine (T4) in Physiological Ranges Using Aptamer-Functionalized Silicon Nanowire Field Effect Transistors

**DOI:** 10.3390/bios16050274

**Published:** 2026-05-09

**Authors:** Stephanie Klinghammer, Wiana Butko, Alexandra Parichenko, Gylxhane Kastrati, Abdallh Herbawi, Leif Riemenschneider, Gianaurelio Cuniberti

**Affiliations:** 1 Institute for Materials Science and Max Bergmann Center for Biomaterials, TU Dresden, 01069 Dresden, Germany; 2 Institute of Biomaterials and Biomolecular Systems, University of Stuttgart, 70569 Stuttgart, Germany; 3 Department of Biological and Biochemical Sciences, Faculty of Chemical Technology, University of Pardubice Studentska 573, 53210 Pardubice, Czech Republic; 4 Cluster of Excellence CARE, TU Dresden and RWTH Aachen, Germany; 5 Cluster of Excellence CeTI, TU Dresden, 01069 Dresden, Germany

**Keywords:** thyroxine, silicon nanowire FET, aptamer biosensor, label-free detection, point-of-care, real-time sensing

## Abstract

Thyroxine (T4) is a key hormone regulating metabolic, cardiovascular, and neurodevelopmental processes, yet its clinical quantification still relies on centralized immunoassays that limit rapid or point-of-care monitoring. Here, we present a label-free biosensing platform based on silicon nanowire field-effect transistors (SiNW-FETs) functionalized with a T4-selective DNA aptamer via a 3-Triethoxysilyl propylsuccinic Anhydride (TESPSA)-mediated silanization approach, enabling a streamlined two-step modification for oriented immobilization. The biosensor achieves robust real-time detection of T4 across the physiological concentration range (5–30 pM), with a limit of detection of ~5 pM and a strong linear correlation between drain current and analyte concentration (R^2^ = 0.9931). Specificity is confirmed using non-functionalized devices and estradiol as a non-target control. All measurements were performed in undiluted phosphate-buffered saline, representing a physiologically relevant ionic environment and demonstrating stable sensor performance under realistic buffer conditions. The dose–response behavior follows a Hill model, allowing extraction of binding parameters and confirming that the electrical signal originates from specific aptamer–target interactions. These results demonstrate that aptamer-functionalized SiNW-FETs provide a highly sensitive, selective, and miniaturizable platform for quantitative thyroid hormone monitoring, with strong potential for future point-of-care applications.

## 1. Introduction

Thyroxine (T4) is one of the primary hormones secreted by the thyroid gland, functioning both as a key regulator of metabolic processes and as a prohormone for the biologically active triiodothyronine (T3) [1]. These hormones orchestrate the activity of multiple organ systems, including cardiovascular, skeletal, and neurological systems, and play crucial roles in thermoregulation, energy homeostasis, and neurodevelopment. In particular, T4 is essential during pregnancy, as maternal thyroid hormone levels directly influence fetal brain development and neurocognitive outcomes [2]. Deviations from normal T4 concentrations can manifest in a wide spectrum of clinical symptoms, which vary depending on whether hormone levels are insufficient (hypothyroidism) or excessive (hyperthyroidism). The physiological concentration of free T4 in human serum ranges from approximately 11.6 to 21.9 pM, with research studies often using a simplified range of 0–25 pM to accommodate variability.

Accurate monitoring of T4 is therefore essential for diagnosis and disease management, particularly during pregnancy, where dysregulated hormone levels can adversely affect fetal development [3].

Accurate quantification of free T4 is therefore essential for diagnosis and management of thyroid disorders. Clinical laboratories routinely employ immunoassays to estimate free T4 levels indirectly. While they are considered faster and more automated than methods such as equilibrium dialysis or LC-MS/MS, immunoassays cannot fully separate T4 from protein-bound forms, and results vary across platforms due to differences in binding protein properties and lack of global standardization [4,5,6]. Consequently, no single method reliably quantifies serum free T4, and clinical interpretation must consider not only T4 levels but also T3, thyroid-stimulating hormone (TSH), as well as patient symptoms. Further, discrepant results often necessitate additional confirmatory assays [7,8].

These limitations have driven growing interest in alternative sensing strategies for rapid, label-free, and potentially continuous biomolecule detection. Biosensors offer a promising solution by enabling direct and often real-time readout of analytes. In particular, point-of-care (POC) platforms support decentralized testing and frequent monitoring through device miniaturization and integration with technologies such as microfluidics [9,10].

Research has actively pursued the development of point-of-care thyroxine (T4) biosensors, with the goal of creating sensitive, selective, and potentially wearable devices. Various strategies have been explored, including amperometric sensors using graphene or gold nanoparticles [11], molecularly imprinted polymer (MIP)-based devices [12], electrochemical immunoassays capable of simultaneous T4 and TSH detection [13], and aptamer-functionalized electrodes [14,15].

Many of these systems achieve very low limits of detection (LODs) in controlled buffer environments—sometimes down to 0.17 pM, well below the physiological T4 range (~10–25 pM)—but their performance in physiologically relevant fluids such as serum or plasma is often suboptimal. This discrepancy arises primarily from the low concentration of free T4 in blood, its high degree of protein binding, and the electrostatic screening effect of ions in biological fluids [16].

Challenges in designing point-of-care thyroxine biosensors arise primarily from the low concentrations of free thyroxine in biological fluids, where most hormone exists in an inactive, protein-bound form. Among the different biosensor types, nanostructured field-effect transistors (FETs) are particularly promising due to their high surface-to-volume ratio. In these devices, changes in surface charge induced by biomolecular binding are directly transduced into electrical signals, enabling label-free, real-time detection without the need for additional amplification steps. Their compatibility with miniaturization and integration into microfluidic or lab-on-a-chip systems further supports point-of-care applications [17,18,19].

Within this class, Silicon nanowire field-effect transistors (SiNW-FETs) have emerged as a highly promising platform. As a result, SiNW-FETs have demonstrated broad applicability across biomedical sensing, including proteins, nucleic acids, ions, neurotransmitters, viruses, bacteria, and cells, with reported sensitivities reaching the attomolar to femtomolar range [20,21,22,23,24].

Despite these advantages, translating FET-based biosensors to physiologically relevant conditions is limited by the Debye screening effect. The Debye length—the characteristic distance over which electrostatic interactions are screened in an ionic solution—shrinks to sub-nanometer scales in high-ionic-strength media such as serum or sweat (~150 mM), which severely restricts the ability of conventional bioreceptors to influence channel conductance [25,26,27].

Several strategies address the Debye limitation [28,29]. Aptamers, short single-stranded DNA or RNA oligonucleotides (<100 nucleotides), offer high-affinity and specific target recognition [30]. Aptamers undergo ligand-induced folding that repositions their charged backbone toward the FET surface, enabling signal transduction within the Debye screening length even in high-ionic-strength environments [31]. Aptamers also provide chemical and thermal stability, resistance to nucleases, and customizable recognition sites. In SiNW-FETs, target binding induces stem-loop folding, bringing charged nucleotides within the effective Debye length, enabling detection regardless of the hormone’s net charge in close vicinity to the FET surface [21,32,33].

Compact bioreceptors such as nanobodies and sybodies—single-domain antibody fragments roughly 2–3 nm in size—have proven effective in high-salt physiological solutions [34]. Interface engineering strategies, including stimuli-responsive polymer brushes [35], hydrogel coverings [36], PEG coatings [37], or electronic double-layer perturbation [38], further enhance sensitivity and prevent nonspecific adsorption.

Building on these properties, aptamer-functionalized SiNW-FETs are particularly suited for selective hormone detection in physiological environments.

In this study, we present a label-free SiNW-FET biosensor functionalized with a thyroxine-specific DNA aptamer for quantitative, real-time detection of free T4 across the physiological range (5–30 pM). The specific binding capability of the T4 aptamer was first confirmed using electrochemical impedance spectroscopy (EIS), which showed a concentration-dependent increase in charge transfer resistance upon T4 binding.

This validation confirmed that the aptamer layer was properly immobilized and functionally active, ensuring that subsequent FET measurements would reliably translate molecular recognition events into measurable electrical signals. By leveraging aptamer conformational gating and optimized TESPSA-mediated immobilization, the platform overcomes the constraints imposed by Debye screening in physiological ionic strength buffers and achieves sensitive detection of thyroxine. Although current measurements were performed in PBS, this approach lays a mechanistic foundation for future adaptation to complex biological fluids and supports the development of portable point-of-care or wearable thyroid hormone monitoring devices. The system can detect both free and unbound T4, spanning concentrations up to four times the healthy range, representing a novel integration of aptamer validation via EIS with high-sensitivity SiNW-FET sensing for frequent, decentralized hormone monitoring.

## 2. Materials and Methods

### 2.1. Materials

Silicon nanowire field-effect transistor (SiNW-FET) biosensors were obtained from i-GEST Co., Ltd. (Pohang, Republic of Korea) [39]. Gold electrodes (Linxens OT5c Gold Electrodes PN 9X85204FA) for electrochemical impedance spectroscopy (EIS) were purchased via PalmSens, (Houten, The Netherlands). The thyroxine-specific DNA aptamer was synthesized by biomers.net GmbH (Ulm, Germany) based on a sequence reported previously [40]. The aptamer was modified at the 5′ end with either a C6-amino or C6-thiol group for covalent immobilization, while the 3′ end remained pristine. Prior to any use, DNA aliquots were heated to 95 °C for 5 min and cooled at a rate of 2.3 °C/min to room temperature using a thermocycler (Eppendorf MasterCycler Pro, Hamburg, Germany).

3-triethoxysilylpropyl succinic anhydride (TESPSA) was purchased from abcr GmbH (Karlsruhe, Germany). Thyroxine (T4), cysteamine, and hydrochloric acid (HCl) were obtained from Sigma-Aldrich (Darmstadt, Germany). Potassium hexacyanoferrate (II) (K_4_[Fe(CN)_6_]) and potassium hexacyanoferrate (III) (K_3_[Fe(CN)_6_]), sodium chloride (NaCl), disodium hydrogen phosphate (Na_2_HPO_4_), and potassium dihydrogen phosphate (KH_2_PO_4_) were purchased from Carl Roth (Karlsruhe, Germany). Potassium chloride (KCl) and magnesium chloride (MgCl_2_) were acquired from Merck KGaA (Darmstadt, Germany). Ethanol, methanol, acetone, and isopropanol were purchased from VWR International (Radnor, PA, USA).

Phosphate-buffered saline (PBS) was freshly prepared with 10 mM Na_2_HPO_4_, 137 mM NaCl, 2.7 mM KCl, and 1.8 mM KH_2_PO_4_ with pH 7.4.

Ag/AgCl paste was received from BAS Inc. (Tokyo, Japan).

### 2.2. Electrochemical Impedance Spectroscopy (EIS)

EIS measurements were conducted to confirm the binding interaction between the aptamer and its analyte thyroxine (T4). Therefore, screen-printed gold electrodes with a gold working electrode (WE), gold Counter electrode (CE) and a Ag/AgCl reference electrode were applied. After cleaning with isopropanol and DI water, followed by drying under a nitrogen (N_2_) stream, RE was coated manually with Ag/AgCl paste and cured at 120 °C for 20 min.

Only the WEs were modified to enable aptamer immobilization: a 10 mM cysteamine solution was applied and incubated for 1 h at room temperature to form a self-assembled monolayer. Next, 5 μM thiolated DNA aptamer solution was drop-cast onto the cysteamine-modified surface. The functionalized electrodes were stored overnight at 4 °C to allow for complete immobilization and were rinsed gently with DI water prior to further use.

EIS measurements were conducted using a PalmSens4 potentiostat equipped with a MUX8-R2 Multiplexer (both PalmSens BV, Houten, The Netherlands) multiplexer and controlled via PS Trace software, Version 5.8.1704. The initial characterization of the electrode surface was performed using cyclic voltammetry (CV) to determine the appropriate DC voltage for EIS measurements.

After each modification step, EIS experiment, impedance spectrum was recorded over a frequency range of 0.1 Hz to 100 kHz with an AC amplitude of 10 mV and the DC voltage determined from the CV analysis.

Further, the functionalized electrode was incubated with T4 solutions of increasing concentrations, ranging from 30 pM, 75 pM, and 150 pM, for 15 min each. After incubation, the electrode was rinsed gently with DI water to remove unbound analytes, dried with nitrogen gas and EIS was recorded.

All EIS measurements were performed in a 5 mM Fe(CN)_6_]/K_3_- and [Fe(CN)_6_]/K_4_-electrolyte solution, prepared in 0.1 M KCl.

Control experiments included measurements of T4 on a bare electrode and estradiol (a structurally similar non-target molecule) on an aptamer-functionalized electrode.

Impedance spectra were analyzed by fitting the data to an equivalent circuit model using the PS Trace software. The charge transfer resistance (Rct) was determined for each condition, and its dependence on analyte concentration was evaluated. A concentration-dependent increase in Rct was observed exclusively for thyroxine on the aptamer-functionalized sensor, confirming the specificity and functionality of the aptamer.

### 2.3. Modification of SiNW-FETs

**Figure 1:**
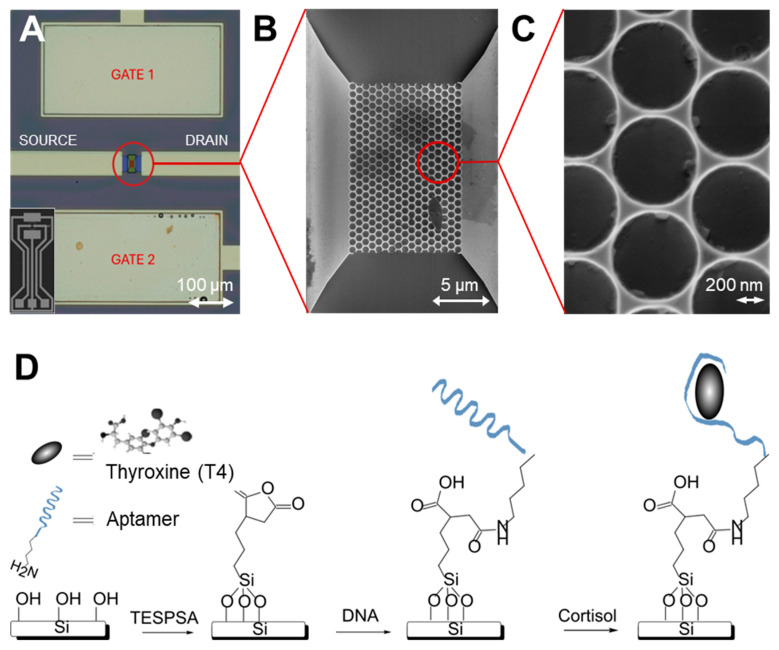
Device geometry and surface functionalization protocol. (**A**) Optical micrograph of the sensor chip layout showing the FET configuration. The central active sensing area is highlighted by the red circle. Sensors provided by i-Gest [39]. (**B**) Scanning electron microscopy (SEM) image of the nanostructured active region. (**C**) Zoom into the nanostructures, illustrating a highly ordered porous. (**D**) Schematic illustration of the stepwise chemical modification of the sensor surface. The process initiates with a hydroxylated silicon surface, followed by silanization with 3-(triethoxysilyl)propylsuccinic anhydride (TESPSA). Subsequently, an amine-terminated DNA aptamer is covalently immobilized via a ring-opening reaction with the succinic anhydride group. The final step involves the specific capture of target molecule Thyroxine (T4).

The honeycomb-structured SiNW-FETs used in this study feature a network geometry that increases the effective surface-to-volume ratio and provides multiple conductive pathways, thereby supporting robust signal transduction. The device is presented in Figure 1A with a respective SEM image of the nanonetwork in Figure 1B,C.

Prior to usage, FETs were thoroughly cleaned by sequential rinsing with deionized (DI) water, ethanol, acetone, and isopropanol, followed by drying with a nitrogen (N_2_) stream. Subsequently, the devices were exposed to oxygen plasma treatment for 10 s at 100 W and 0.3 bar (Zepto One, Diener electronic GmbH & Co. KG, Ebhausen, Germany) to hydroxylate the surface.

Directly following this, a straightforward two-step TESPSA-based silanization protocol is employed for aptamer immobilization to replace conventional multi-step NHS/EDC coupling chemistry. Surface salinization using TESPSA and further with amino-modified DNA aptamers was performed as described by Gang et al. [41] and sketched schematically in Figure 1D. Briefly, the activated surfaces were incubated under vacuum and infrared heating with 100 µL TESPSA for 4 h. To stabilize the silane layer and prevent undesired ring-opening reactions of the anhydride, the samples were cured at 120 °C for 30 min. Following silanization, 5 µM amino-modified DNA aptamers were prepared in 1× PBS, supplemented with 5 mM MgCl_2_. The TESPSA-functionalized surfaces were incubated with the aptamer solution for 1 h at room temperature to enable covalent attachment via amide bond formation. After incubation, the sensors were rinsed thoroughly with PBS and DI water to remove unbound aptamers. The samples were stored at 4 °C until further use.

### 2.4. SiNW-FET Setup

Electrical measurements were conducted using a Keithley 2604B SourceMeter (Keithley Instruments, a Tektronix Company, Cleveland, OH, USA). During transfer curve measurements, the gate voltage (VG) was incrementally swept from 0 to +2 V with 0.05 V steps, while the source–drain voltage (VDS) was maintained at a constant +0.1 V. For real-time experiments, VG was held at a fixed value corresponding to the linear region of the transfer curve, ensuring stable and reliable measurements.

### 2.5. Thyroxine Detection

In analogy to EIS measurements, T4 solutions were prepared at concentrations ranging from 5 pM to 30 pM, spanning the physiological range of free thyroxine in blood serum. For each concentration, 30 µL of the solution was drop-cast onto the sensor surface and incubated for 5 min before rinsing with PBS. Transfer characteristics and real-time current responses were recorded after each incubation. Additional measurements were performed at higher T4 concentrations (100–600 pM) to determine the saturation point and calculate the dissociation constant (Kd) using the Hill equation.

The limit of detection (LOD) was estimated based on the signal-to-noise characteristics of the baseline response and the slope of the corresponding calibration curve, following a standard approach commonly used for biosensing systems.

### 2.6. Data Analysis

Current values were extracted and analyzed using MATLAB R2016b. For real-time measurements, the average current during PBS washing steps was calculated to determine concentration-dependent shifts. The Hill equation was used to model the dose–response curve and extract relevant binding parameters. Specificity was assessed by comparing the current response for T4 and non-target estradiol.

Data are reported as mean ± standard deviation based on at least three independent experiments, unless stated otherwise. For clarity of presentation, representative datasets are shown for time-resolved and real-time measurements, while full datasets are provided in summary plots. Variability between measurements is indicated using error bars in all graphical representations.

## 3. Results

### 3.1. Electrochemical Validation of Aptamer–Target Interaction

Electrochemical impedance spectroscopy (EIS) and cyclic voltammetry (CV) were employed to verify the binding capability and specificity of the T4-specific DNA aptamer prior to SiNW-FET experiments. CV measurements characterized the electrode surface and confirmed aptamer immobilization (Figure S1A): peak currents decreased after aptamer functionalization and further upon incubation with 150 pM T4, indicating successful formation of the aptamer–target complex.

**Figure 2:**
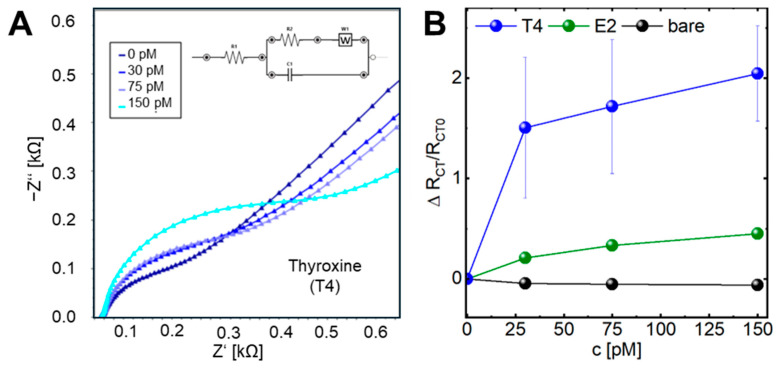
Electrochemical impedance spectroscopy (EIS) analysis of aptamer–analyte interactions. (**A**) Nyquist plots show the charge transfer resistance (Rct) at the electrode interface for increasing concentrations of T4 (0, 30, 75, 150 pM) and (**B**) Normalized charge transfer resistance (Rct/R_0_) extracted from EIS measurements as a function of analyte concentration. Inset in A depicts the equivalent circuit used for fitting the EIS data.

EIS measurements quantified changes in charge transfer resistance (Rct) during each modification step. The bare electrode exhibited an Rct of ~0.9 kΩ, increasing to 1.2 kΩ after aptamer immobilization (Figure S1B). Upon incubation with the target analyte T4 (30, 75, 150 pM), Rct increased to 1.8, 2.4, and 3.1 kΩ, respectively, showing a clear concentration-dependent response (Figure 2A). To account for device-to-device variability, charge transfer resistance values were normalized to the corresponding baseline (R_0_), enabling direct comparison across independently fabricated electrodes.

Here, Figure 2B summarizes the concentration-dependent normalized charge transfer resistance (Rct/R_0_) extracted from equivalent circuit fitting of the respective EIS spectra for T4, the control E2 (compare to Figure S1C) and untreated sensors. This normalization highlights relative interfacial changes rather than absolute resistance values, which can vary between individual electrodes due to fabrication-induced variability. The pronounced increase in Rct/R_0_ upon T4 exposure, compared to minimal changes observed for control conditions, confirms that the impedance response originates from specific aptamer–target interactions rather than nonspecific adsorption or electrode variability.

This behavior is consistent with T4-induced aptamer conformational changes, which increase interfacial layer density and hinder redox probe access to the electrode surface [42]. Minor deviations from expected trends, such as lower Rct on some functionalized sensors, can be explained by incomplete blocking or sparse coverage of flexible aptamers. Control experiments using bare electrodes and estradiol confirm that the observed impedance changes are primarily driven by specific T4–aptamer interactions, while only minor nonspecific variations are observed.

Importantly, EIS and SiNW-FET measurements probe the same fundamental interfacial event—namely target-induced changes in the electrical double layer at the sensor surface—but differ in their transduction mechanisms, with EIS reflecting impedance modulation at the electrode interface and the FET translating local surface charge variations into amplified conductance changes. Therefore, the relationship between both techniques is conceptual rather than quantitative.

These results confirm that the chosen aptamer is capable of specific, quantitative detection of T4. While EIS validates aptamer functionality, its limitations in real-time monitoring and miniaturization highlight the need for integration with FET-based platforms. This electrochemical validation provides a strong foundation for SiNW-FET sensing.

### 3.2. Detection of T4 with SiNW-FETs

#### 3.2.1. Device Validation

The functionality of the SiNW-FET platform was verified through evaluation of the pH sensitivity of the nanowire channel. The drain current was recorded at a constant bias voltage while the pH of the measurement buffer was systematically varied. A clear and reproducible change in the drain current was observed with increasing pH, indicating that the device is responsive to variations in surface charge at the nanowire–electrolyte interface. The corresponding pH response curves are provided in the Supporting Information (Figure S2). The observed current modulation was calculated as −16.24nA/pH, which is slightly lower than reported values in literature and can be assumed to be dependent on the surface chemistry of the nanowires, the effective gate coupling, and the nanowire network geometry and buffer ionic strength, which together determine transductance of individual devices into drain current changes [43,44,45]. This sensitivity to local charge changes is a key prerequisite for biosensing, as molecular binding events at the functionalized nanowire surface directly alter the electrostatic environment [46]. These results validate the platform for thyroxine detection by demonstrating reliable transduction of surface charge changes into electrical signals.

#### 3.2.2. Electrical Detection of T4

**Figure 3:**
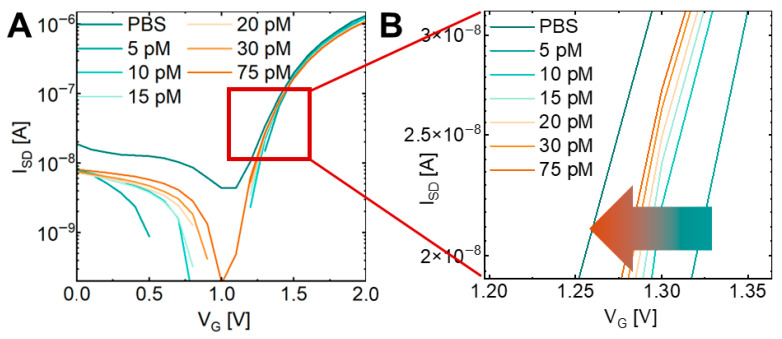
Electrical response of the SiNW-FET biosensor to varying concentrations of T4. (**A**) Transfer characteristics recorded in PBS (control) and upon exposure to T4 concentrations ranging from 5 pM to 75 pM. (**B**) Magnified view of the subthreshold region highlighted by the red box in (**A**). The arrow indicates a progressive shift in the threshold voltage with increasing analyte concentrations, demonstrating the field-effect-based detection mechanism.

Following validation of device functionality, the aptamer-functionalized SiNW-FET was applied to the detection of thyroxine (T4). The T4-specific DNA aptamer was covalently immobilized on the nanowire surface via TESPSA-mediated silanization, providing stable orientation and efficient target recognition for subsequent electrical measurements. Initial characterization was performed using discrete I–V curves, with the drain current recorded over a range of gate voltages for each T4 concentration (Figure 3). These measurements revealed clear, concentration-dependent shifts in the I–V characteristics, particularly beyond the threshold voltage of ~1.4 V, confirming that T4 binding to the immobilized aptamer modulates the local electrostatic environment at the nanowire surface and alters channel conductance. The I–V analysis further enabled identification of an optimal operating point for subsequent measurements.

For continuous real-time monitoring, two approaches are possible. Continuous I–V sweeping allows enhanced sensitivity and a comprehensive overview of sensor response [47] but it requires higher gate voltages, which can induce Joule heating, material degradation, and disturbed electrokinetic flow, compromising device integrity and long-term stability [48,49].

**Figure 4:**
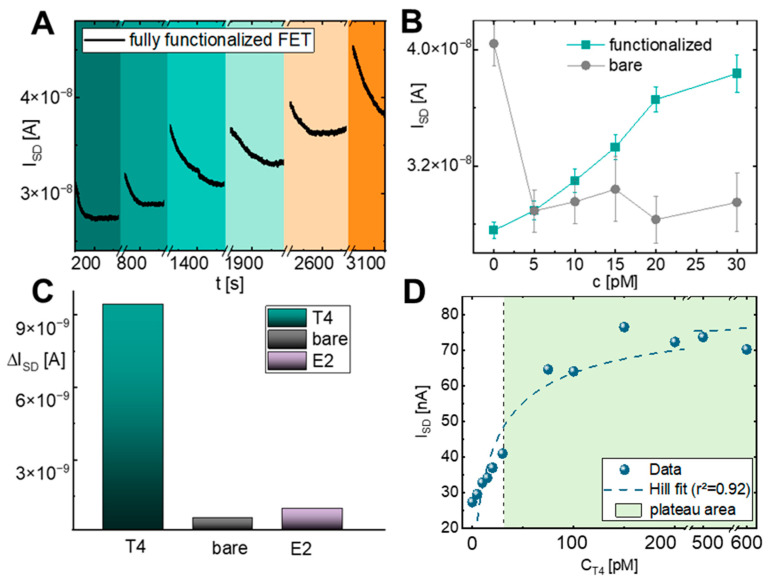
Performance and characterization of the functionalized FET biosensor. (**A**) Real-time recording during sequential exposure to increasing T4 concentrations. (**B**) Calibration curves comparing the response of functionalized (petrol) versus bare (grey) devices across a T4 concentration range from 0 to 30 pM. (**C**) Comparison of the current response for the target analyte (T4), non-functionalized sensors, and the non-target molecule estradiol (E2). While signal variations are observed in all cases, T4 induces a significantly stronger and more consistent concentration-dependent response compared to control measurements. (**D**) Dose–response curve for T4 concentration ranging from 0 to 600 pM. The experimental data (blue circles) are fitted with a Hill equation (dashed line), highlighting the sensor’s saturation in the plateau area.

To preserve device function while maintaining reliable detection, sensors were operated at a fixed gate voltage of 1.4 V, determined from discrete I–V analysis. Under these conditions, the aptamer-functionalized SiNW-FETs enabled real-time current monitoring of T4 binding, as shown in Figure 4A. For clarity of quantitative evaluation, signal analysis focuses on stabilized response regions, particularly after rinsing, while the full real-time current traces during incubation and washing are provided in the Supporting Information (Figure S3). Signals during incubation differ from those after rinsing due to the presence of unbound target molecules, whereas rinsing yields a stabilized response used for quantitative evaluation.

From these continuously recorded current traces, the dose-dependent response was extracted and is summarized in Figure 4B, revealing reproducible, near-linear increases in drain current across the physiologically relevant range (R^2^ = 0.9931) with an average sensitivity of 2.31 nA/pM. This fixed-voltage, continuous monitoring approach ensures stable, real-time detection while minimizing electrical stress, which is critical for point-of-care applications.

Control experiments confirmed the specificity of the response, as summarized in Figure 4C. Bare (unmodified) sensors showed no meaningful correlation between current and T4 concentration. Further, measurements with estradiol on functionalized sensors also resulted in signal variations; however, these were significantly smaller in magnitude and did not exhibit a consistent concentration-dependent trend. The real time graphs, from which the presented changes were extracted from, can be found in Figures S4 and S5 respectively. These findings suggest that the observed response is primarily driven by T4–aptamer interactions, while nonspecific contributions appear to be minor.

To quantitatively describe the binding behavior, the concentration-dependent response was analyzed using a Hill model (Figure 4D). The fit yielded an apparent dissociation constant (K_D_) of 43.96 pM, indicating high affinity of the aptamer for T4. The corresponding logistic fit revealed a saturation plateau at ~90.23 pM, consistent with full occupancy of available binding sites. This analysis not only supports the specificity and sensitivity of the sensor but also highlights its applicability for quantitative detection within and beyond the physiological T4 range.

It should be noted that signal variations can arise from solution exchange processes and intrinsic device-to-device variability. While these effects contribute to baseline fluctuations, they do not obscure the overall concentration-dependent trend observed for T4.

The dose-dependent response originates from aptamer conformational changes, where target binding induces structural rearrangements that bring charged phosphate groups closer to the nanowire surface, enabling modulation of the local electric field under ionic screening conditions. While techniques such as Förster resonance energy transfer [50,51] and circular dichroism [52] have been used to probe aptamer conformational changes, EIS was chosen here for its direct sensitivity to interfacial electrical changes and hence straightforward method to confirm electrical changes at the electrode interface. The specificity of this interaction was independently supported by electrochemical impedance spectroscopy (Section 3.1), which showed a concentration-dependent increase in charge transfer resistance upon T4 binding. While EIS provides high sensitivity for validating molecular interactions, its limited compatibility with miniaturized and real-time platforms highlights the advantages of SiNW-FETs. Their high surface-to-volume ratio, label-free operation, and compatibility with scalable device architectures make them particularly well suited for real-time, quantitative biosensing in point-of-care applications.

Taken together, these results demonstrate that the SiNW-FET platform reliably converts T4 concentration-dependent binding events into measurable current variations in a real-time measurement format. Aptamer functionalization provides enhanced sensitivity and indicates target-selective response characteristics under the investigated conditions. The combined workflow—from pH validation to discrete I–V characterization and fixed-voltage monitoring—establishes a robust and device-preserving strategy for rapid and selective hormone detection.

The constructed SiNW-FET biosensor demonstrates strong potential for quantitative thyroxine detection in physiologically relevant concentrations. Compared to conventional methods, this approach offers label-free operation, rapid analysis, and high selectivity. Further improvements in microfluidic integration and validation in complex biological fluids will be essential for translation into wearable and point-of-care applications.

## 4. Conclusions

We present a fully label-free, aptamer-functionalized SiNW-FET platform for the quantitative detection of free thyroxine (T4) within and below the physiological range. Electrochemical validation via EIS confirmed specific, concentration-dependent aptamer–T4 interactions, while real-time FET measurements transduced these binding events into reproducible electrical signals with high sensitivity (2.31 nA/pM) and a near-linear response across physiologically relevant concentrations. The observed signal arises from aptamer conformational switching, which brings negatively charged phosphate groups into proximity with the nanowire surface, modulating the local electric field within the Debye length. This mechanism, combined with TESPSA-mediated oriented immobilization, enables effective signal transduction in high-ionic-strength buffers, consistent with partial localization of charge perturbations within the effective Debye length.

Control experiments indicate a preferential response toward the target analyte compared to non-target molecules, and the dose–response behavior was well-described by the Hill model, yielding a dissociation constant (K_d) of 43.96 pM. Compared to traditional immunoassays, this approach offers rapid, label-free, and real-time sensing, with potential for miniaturization and integration into point-of-care compatible device architectures. The workflow—spanning aptamer validation, discrete I–V characterization, and fixed-voltage monitoring—establishes a robust proof-of-concept strategy for sensitive and selective hormone detection under controlled conditions.

Current limitations include operation in buffer-only environments, the absence of long-term stability studies, and the lack of integration with microfluidic and portable readout electronics, which will be addressed in future work through evaluation in complex biological fluids and integration with microfluidic and electronic readout components to further assess applicability toward decentralized thyroid hormone monitoring.

## Data Availability

The data that support the findings of this study are available from the corresponding author upon reasonable request.

## Supplementary Materials

The following supporting information can be downloaded at: https://www.mdpi.com/article/10.3390/bios16050274/s1, Figure S1: (A) Cyclic voltammograms of the electrode before (green) and after (orange) immobilization. (B) EIS measurements of bare and aptamer functionalized sensor. (C) Nyquist plot of fully functionalized sensor against control estradiol (E2); Figure S2: pH-dependent drain current response of the SiNW-FET measured at fixed gate voltage (pH 6.0–8.0), demon-strating reproducible sensitivity to surface charge variations. (A) real-time response and (B) extracted currents for each pH value; Figure S3: Real-time drain current response of the aptamer-functionalized SiNW-FET during stepwise T4 incubation, showing continuous signal evolution and stabilization upon binding equilibrium; Figure S4: Real-time drain current response of the non-functionalized SiNW-FET during stepwise T4 incubation; Figure S5: Real-time drain current response of the aptamer-functionalized SiNW-FET during stepwise estradiol incubation.
