# Supplementary material for: Ultrasensitive Label-Free Detection of Free Thyroxine (T4) in Physiological Ranges Using Aptamer-Functionalized Silicon Nanowire Field Effect Transistors
Source: Biosensors (Basel). 2026 May 9;16(5):274. doi: 10.3390/bios16050274 (PMC13205116; doi:10.3390/bios16050274)
Supplement: Supplementary file 1 [file biosensors-16-00274-s001.zip › biosensors-4262990-supplementary.pdf]

biosensors

MDPI

Supporting Information to the article

Ultrasensitive Label-Free Detection of Free Thyroxine (T4) in Physiological Ranges Using Aptamer-Functionalized Silicon Nanowire Field Effect Transistors

Stephanie Klinghammer [1,*,†], Wiana Butko [1,2,†], Alexandra Parichenko [1], Gylxhane Kastrati [1,3], Abdallh Herbawi [1], Leif Riemenschneider [1] and Gianaurelio Cuniberti [1,4,5,*]

[1] Institute for Materials Science and Max Bergmann Center for Biomaterials, TU Dresden, 01069 Dresden, Germany
[2] Institute of Biomaterials and Biomolecular Systems, University of Stuttgart, 70569 Stuttgart, Germany
[3] Department of Biological and Biochemical Sciences, Faculty of Chemical Technology, University of Pardubice Studentska 573, 53210 Pardubice, Czech Republic
[4] Cluster of Excellence CARE, TU Dresden and RWTH Aachen, Germany
[5] Cluster of Excellence CeTI, TU Dresden, 01069 Dresden, Germany
* Correspondence: stephanie.klinghammer@tu-dresden.de (S.K.); gianaurelio.cuniberti@tu-dresden.de (G.C.)
† These authors contributed equally to this work.

1. Electrochemical Validation of Aptamer–Target Interaction

Cyclic voltammograms of the electrode surface before (green) and after (orange) immobilization of the biosensing layer are presented. The modification leads to a clear change in peak shape and current response, indicating successful surface functionalization. The shift and attenuation of the redox peaks after immobilization reflect hindered electron transfer due to the formation of the sensing layer. Based on these results, the potential corresponding to the oxidation peak was selected for subsequent electrochemical impedance spectroscopy (EIS) measurements (figure S1B). The corresponding EIS spectra of sensors exposed to various concentrations of the control E2 are presented in Figure S1C.

 https://doi.org/10.3390/bios16050274

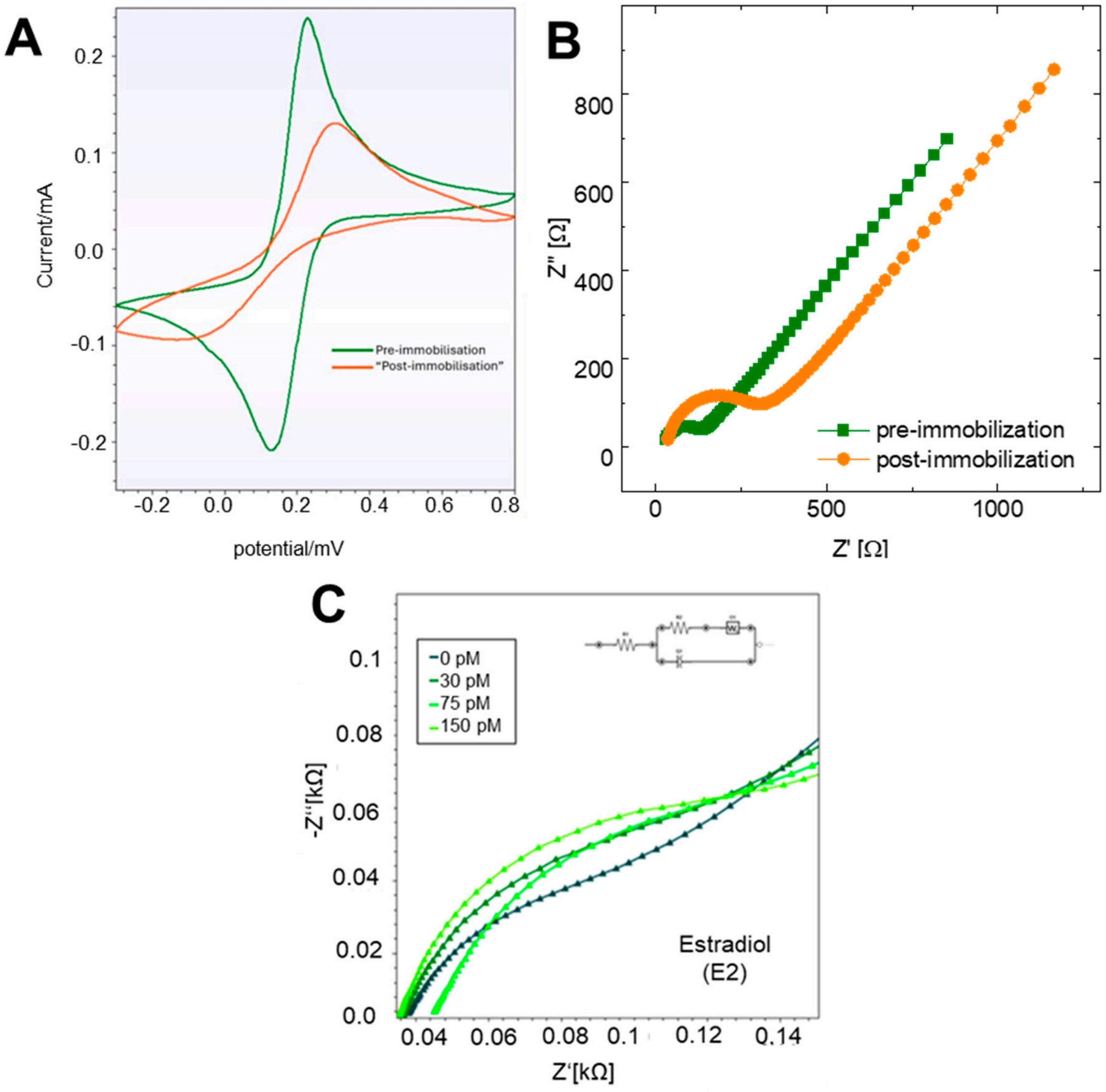

Figure S1. (A) Cyclic voltammograms of the electrode before (green) and after (orange) immobilization. (B) EIS measurements of bare and aptamer functionalized sensor. (C) Nyquist plot of fully functionalized sensor against control estradiol (E2).

2. Device validation through pH measurements

The pH sensitivity of the SiNW-FET devices was evaluated to confirm proper transistor operation and responsiveness to surface charge variations. Measurements were performed by recording the drain current at a fixed gate voltage while systematically varying the pH of the buffer solution from pH 6.0 to 8.0 (Figure S2).

The real-time response (Figure S2A) shows a clear and reproducible decrease in drain current with increasing pH, consistent with deprotonation of surface silanol groups and the resulting modulation of the surface potential. This behavior reflects the intrinsic sensitivity of the nanowire channel to changes in the interfacial charge environment.

The extracted steady-state current values (Figure S2B) reveal a monotonic pH-dependent trend, from which a sensitivity of −16.24 nA/pH was determined. This value is slightly lower than the ideal Nernstian response but within the expected range for nanowire-based FET sensors. The deviation can be attributed to factors such as surface functionalization, buffer ionic strength, and device geometry, which influence effective gate coupling.

https://doi.org/10.3390/bios16050274

These results confirm stable device operation and demonstrate the capability of the SiNW-FET platform to transduce electrostatic changes at the solid–liquid interface into measurable electrical signals, providing a reliable basis for subsequent biosensing experiments.

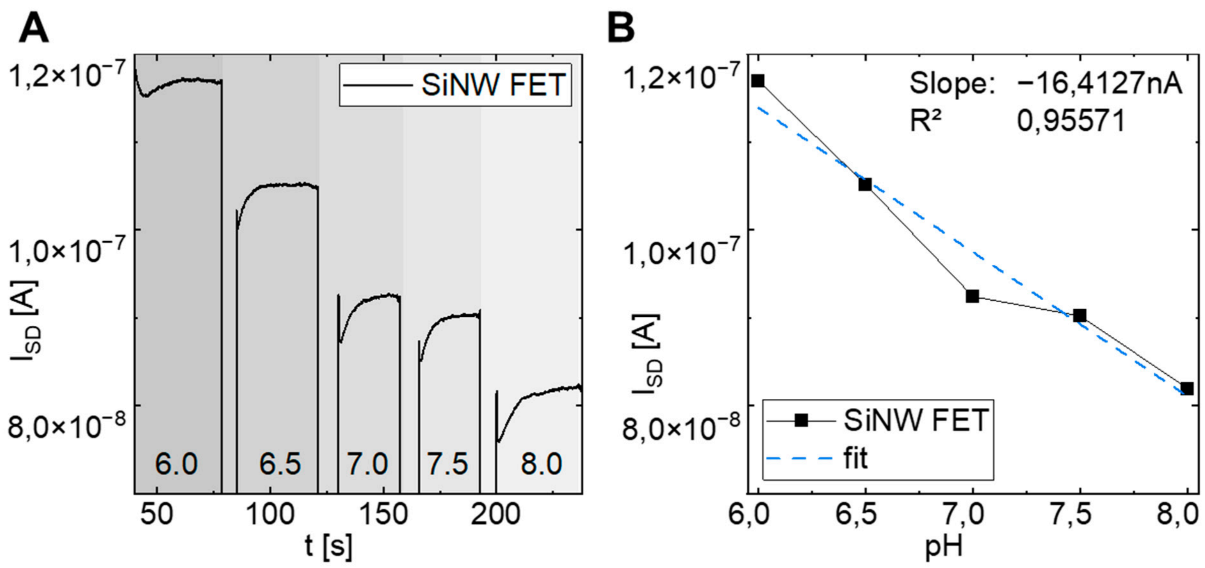

Figure S2. pH-dependent drain current response of the SiNW-FET measured at fixed gate voltage (pH 6.0–8.0), demonstrating reproducible sensitivity to surface charge variations. A) real-time response and B) extracted currents for each pH value.

3. Real-Time Signal Evolution During T4 Incubation

To evaluate the temporal evolution of the sensor response, full real-time recordings were acquired during T4 incubation (Figure S3). In contrast to the main manuscript, where only the stabilized signal levels are presented, these measurements capture the complete binding process.

A continuous change in drain current was observed throughout the incubation period, reflecting the dynamic interaction between the T4 molecules and the immobilized aptamer layer. The signal gradually evolved before reaching a quasi-stable plateau, indicating that binding equilibrium had been established.

These results demonstrate that while the sensor response is time-dependent during incubation, reliable and reproducible detection can be achieved once the signal stabilizes. This justifies the use of defined measurement time points in the main manuscript and confirms that the reported values correspond to equilibrium conditions suitable for quantitative analysis.

https://doi.org/10.3390/bios16050274

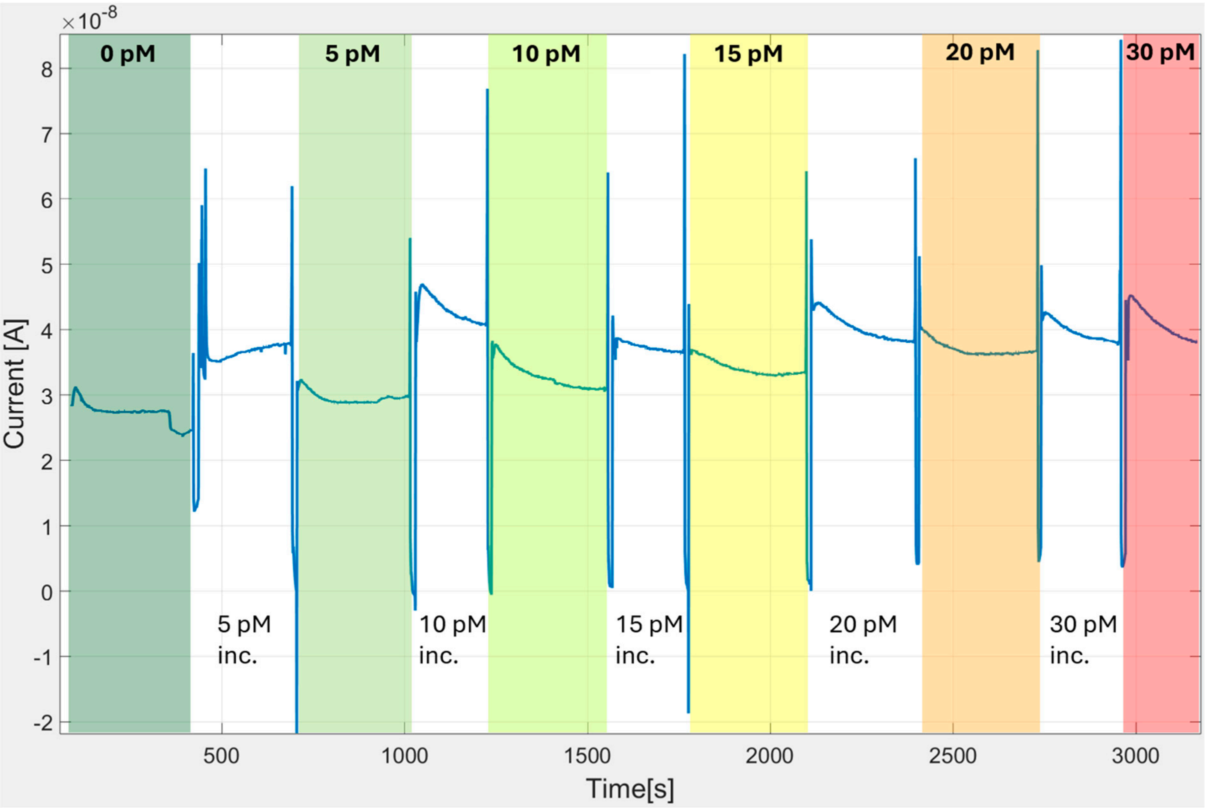

Figure S3. Real-time drain current response of the aptamer-functionalized SiNW-FET during stepwise T4 incubation, showing continuous signal evolution and stabilization upon binding equilibrium.

To validate the specificity of the sensor response, real-time measurements of control experiments are provided in Figures S4 and S5. Figure S4 shows the response of non-functionalized (bare) SiNW-FET devices during T4 exposure, while Figure S5 presents measurements of aptamer-functionalized sensors exposed to estradiol.

In both cases, no clear concentration-dependent signal change or stable plateau formation was observed. The recorded current fluctuations remain within the baseline variation, indicating the absence of specific binding-induced modulation of the nanowire channel.

These full real-time datasets form the basis for the extracted values presented in Figure 3c of the main manuscript and confirm that the observed sensor response is specific to the interaction between thyroxine and the immobilized aptamer.

https://doi.org/10.3390/bios16050274

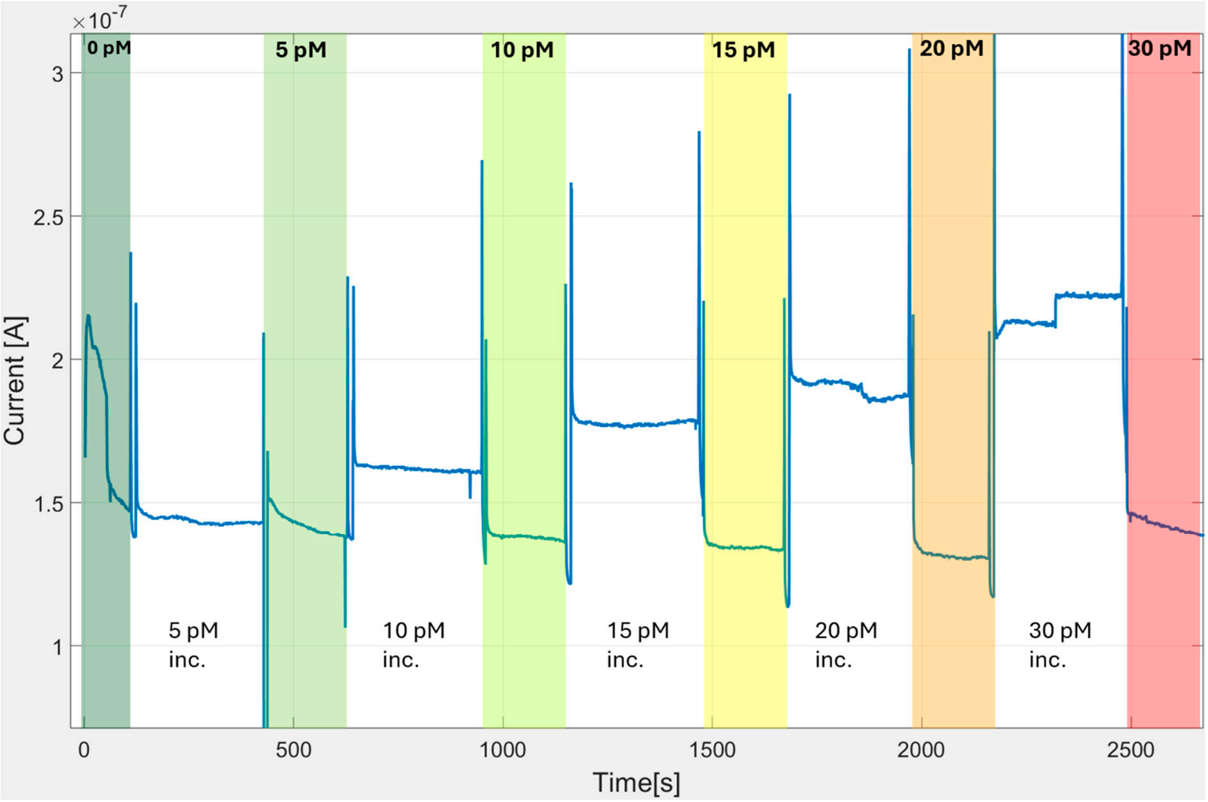

Figure S4. Real-time drain current response of the non-functionalized SiNW-FET during stepwise T4 incubation.

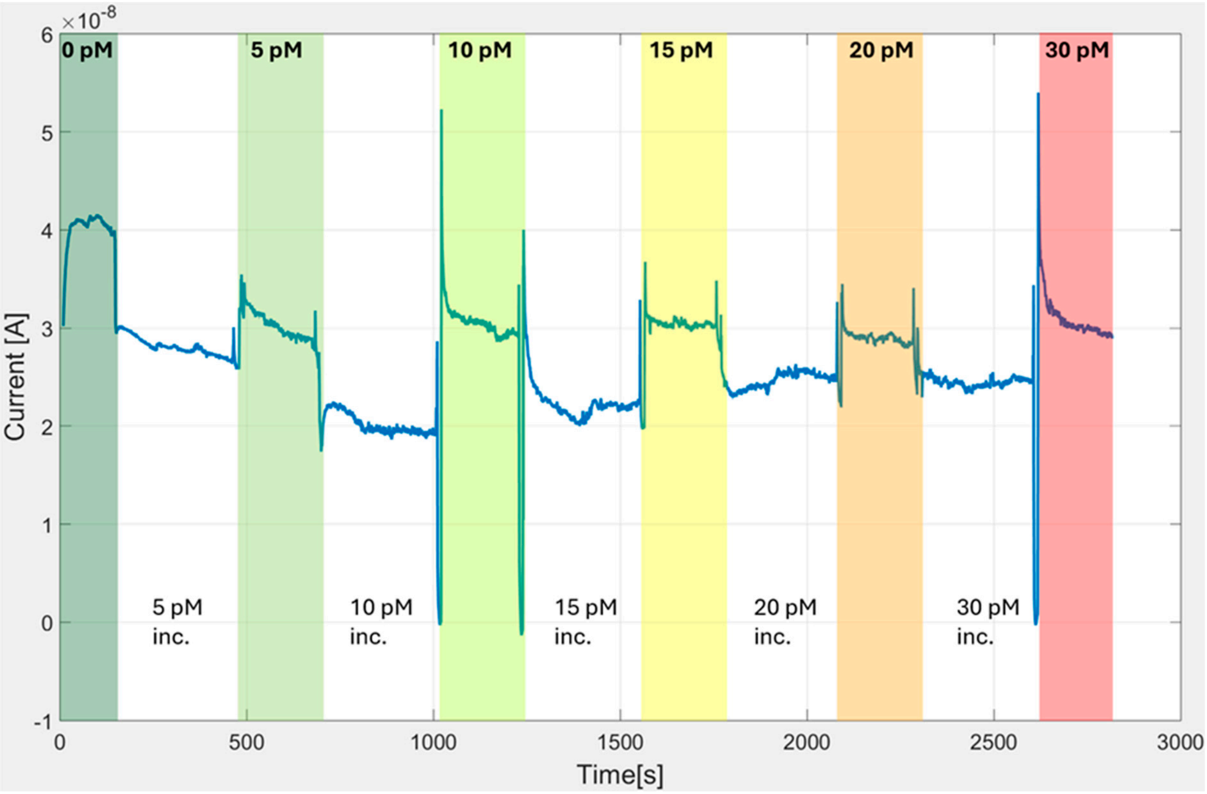

Figure S5. Real-time drain current response of the aptamer-functionalized SiNW-FET during stepwise estradiol incubation.

Disclaimer/Publisher's Note: The statements, opinions and data contained in all publications are solely those of the individual author(s) and contributor(s) and not of MDPI and/or the editor(s). MDPI and/or the editor(s) disclaim responsibility for any injury to people or property resulting from any ideas, methods, instructions or products referred to in the content.

https://doi.org/10.3390/bios16050274
